# Thermal Requirements Underpinning Germination Allude to Risk of Species Decline from Climate Warming

**DOI:** 10.3390/plants9060796

**Published:** 2020-06-25

**Authors:** Jennifer Anne Cochrane

**Affiliations:** 1 Department of Biodiversity, Conservation and Attractions, Locked Bag 104, Bentley Delivery Centre, Western Australia 6983, Australia; anne.cochrane@dbca.wa.gov.au; Tel.: +61-429-698-644; 2 Division of Ecology and Evolution, College of Science, Australian National University, Canberra ACT 0200, Australia

**Keywords:** temperature, seed germination, germination rate, degree-days, global warming

## Abstract

The storage of seeds is a commonly used means of preserving plant genetic diversity in the face of rising threats such as climate change. Here, the findings of research from the past decade into thermal requirements for germination are synthesised for more than 100 plant species from southern Western Australia. This global biodiversity hotspot is predicted to suffer major plant collapse under forecast climate change. A temperature gradient plate was used to assess the thermal requirements underpinning seed germination in both commonly occurring and geographically restricted species. The results suggest that the local climate of the seed source sites does not drive seed responses, neither is it indicative of temperatures for optimal germination. The low diurnal phase of the temperature regime provided the most significant impact on germination timing. Several species germinated optimally at mean temperatures below or close to current wet quarter temperatures, and more than 40% of species were likely to be impacted in the future, with germination occurring under supra-optimal temperature conditions. This research highlights both species vulnerability and resilience to a warming climate during the regeneration phase of the life cycle and provides vital information for those aiming to manage, conserve and restore this regional flora.

## 1. Introduction

In an era of unprecedented biodiversity loss, seed banks have become a vital means to support the conservation of the genetic diversity of flowering plant species [1,2]. The storage of seeds complements plant conservation in the wild and provides the raw material for population augmentation and the creation of new populations in the face of biodiversity decline and loss [3,4]. Seed banks also provide the raw material for research into the germination requirements of species [5,6], including documenting seed responses to a diverse range of threatening processes [7,8,9,10]. The data obtained from comprehensive seed research provides vital knowledge that underpins the use of seeds in plant reintroductions and restoration works and determines whether stored seeds are viable or not. However, complex dormancy mechanisms often hinder a thorough understanding of seed germination without comprehensive testing. Some forms of dormancy (e.g., physical dormancy, PD) are relatively easy to overcome but more complex dormancy mechanisms (e.g., physiological or morphophysiological dormancy) can be problematic, and there is still much research required. Tetrazolium staining and X-ray techniques may provide certain benefits for determining seed viability but are no replacement for the germination test that allows us to create new plants from seeds, which is the most valuable outcome of any seed conservation program. Understanding seed performance under a range of environmental conditions is particularly pertinent in the face of a changing climate [11].

Temperature is one of the most important signals for stimulating germination, providing the seed with both temporal and predictive information [12]. Temperature influences the numbers of seeds that germinate, the rate at which they germinate and the season of germination [13,14]. The rate of germination (reciprocal of the time taken to germinate) in non-dormant seeds shows a positive linear relation between the base temperature (at and below which no germination occurs) and the optimum temperature (at which germination is maximal); and a negative linear relation between the optimal temperature and the ceiling temperature (at and above which germination is again zero). The temperature requirements for seed germination, including the temperature niche width and the rate of germination, are important seed traits that impact on seed functions such as germination timing in the field [15]. Germination timing is under strong natural selection [16,17], but also shows a degree of phenotypic plasticity [18]. The phenotypic expression of post-germination life-history characteristics can be altered by germination timing [19] and can have a large impact on community assembly [20,21,22]. The timing of germination determines the environmental conditions experienced by seedlings and has a major effect on plant fitness, survival, persistence, ecological niches, distribution ranges, and evolutionary potential [17,23]. Early germination can benefit plants through access to resources (nutrients, light, water and space), thereby increasing competitive interactions, whilst late germinating species may increase their risk of exposure to unfavourable conditions [24]. However, recent research has demonstrated improved survival of seedlings of an annual species when neighbouring seedlings are larger [25]. In seasonally dry environments, timing germination to coincide with winter rains enables adequate seedling growth prior to the onset of summer drought [26], as later season drought stress is a common cause of seedling mortality. Germination timing is a crucial function of how plants will respond to a changing climate [27,28], whilst the identification of the temperatures for dormancy release and germination, including a seeds’ thermal time requirements, can provide insight into plant adaptation and ecological strategies in relation to current and future thermal environments [15]. These seed traits are less studied than other plant traits (for example seed mass), but in the context of a warming climate are essential for understanding future plant regeneration.

In the context of a warming climate, the ability of seed-bearing plants to persist in situ will rely on successful seed reproduction, regeneration and colonisation [29]. There is little doubt that global temperatures are rising, becoming more variable and extreme [30]. The majority of studies using thermal time models generally only consider constant temperatures or at best one diurnal temperature cycle for germination (e.g., [31,32,33]) with few exceptions [34,35]. However, temperatures in the natural environment are not constant [36] and alternating temperatures occur in most habitats, with the largest diurnal temperature shifts occurring at higher altitude and latitude. These alternating temperatures provide a mechanism for sensing seasonal shifts, depth of soil burial and may substitute for a light requirement for germination [29].

Rising temperatures are expected to alter post-dispersal seed germination cues [11], leading to a potential loss of plant diversity. In some cases, warmer climates are predicted to increase germination, for example in winter annuals [37] and alpine species [38]; in other cases, it has been suggested that global warming will cause a decrease in the number [39,40] and diversity [11,40] of seedlings recruited in the future. The overall disparity reported between observed and realised thermal niches suggests a high likelihood of underestimating species thermal tolerances. [41,42]. Consequently, climatic requirements for germination beyond those in a species current distribution may need to be examined.

Southern Western Australia is one of the locations predicted to suffer major plant collapse under forecast climate change [43,44,45]. However, a number of recent studies suggest that wild sourced native seeds from this global biodiversity hotspot are relatively resilient to high temperature stress [8,10,46,47,48]. These previous papers have taken a taxonomic, local flora or life form focus to assess the thermal requirements for germination of species from the area. Here, I provide a synthesis of these previous studies which comprise a flora representative of a largely Mediterranean-climate ecosystem with a diverse range of predominantly perennial species. Data from the previous studies and additional unpublished data have been re-analysed to help develop a better understanding of how a particular seed trait (i.e., temperature requirements) impacts on a specific seed function (i.e., germination timing) in a regional flora. With this larger dataset, I have posed several key questions relating to temperature effects on germination: (1) will seeds of native plant species from this flora tolerate a warming climate? (2) will changes to thermal regimes associated with a warming climate alter their germination timing? (3) what feature of the diurnal temperature cycle will impact most on germination? (4) will commonly occurring species with widespread distributions and species with more restricted distributions differ in their response to thermal conditions? My expectations are that species from the northern boundaries of this part of Western Australia will tolerate a warmer climate more so than those inhabiting the southern reaches; that germination timing will vary, with species in the northern part germinating earlier than those in the more southerly latitudes due to the influence of temperature; and that common species will have a wider temperature niche for germination compared to species with a more restricted distribution. This species-rich flora occupies a highly seasonal environment and it is critical to obtain an understanding of germination performance under current conditions as well as predict the same under future conditions.

## 2. Results

Complete germination (i.e., 100%) occurred under at least one diurnal temperature regime in all but 16 of the 113 collections investigated. Seeds of nine of these 16 collections attained at least 90% germination. Only four species (*Borya sphaerocephala*, *Stylidium scandens*, *Xyris lacera* and *X. maxima*) failed to germinate well (<60%) under any temperature regime and these species were deemed to possess dormancy mechanisms not overcome by temperature alone (no pre-treatment was provided prior to incubation in these species).

**Figure 1:**
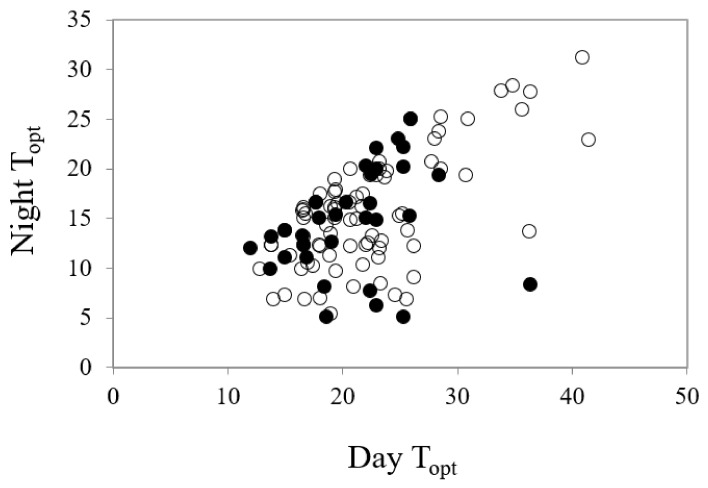
The relationship between the diurnal temperature conditions (°C) required for highest and most rapid germination (T_opt_—optimum temperature for germination) in common (○, r^2^ = 0.4384) and restricted species (●, r^2^ = 0.088) from southern Western Australia.

**Figure 2:**
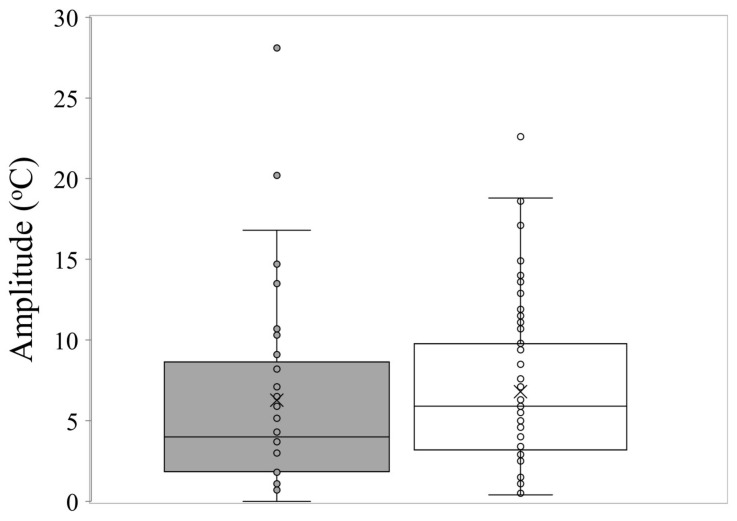
Box and whisker plot showing the amplitude of temperature fluctuations for optimum germination performance in common (□) and restricted species (■) from southern Western Australia. The X represents the mean, the horizontal line is the median, upper and lower boundaries of the box represent the interquartile range (75th and 25th percentiles), and whiskers extend from maximum to minimum.

The mean time to first germination differed little between common and conservation-listed species and was approximately 12 days, with time to reach 50% germination at around 16 days for both categories of plant species. The mean temperature for optimal germination was 18.5 °C across all collections. The majority of commonly occurring species had their optimum germination occurring at an average of 18.8 °C with diurnal ranges between 15–25 °C during the day and 10–20 °C at night. However, there were several exceptions with far greater temperature requirements (e.g., *Eucalyptus erythrocorys* from north of Perth which germinated best at 36 °C). The overall spread of diurnal temperatures providing optimal germination is presented in Figure 1. Table 1 summarises data on the germination parameters (percentage germination, lag time, mean time to germination, germination rate and its reciprocal, temperatures during the diurnal cycle and their amplitude, mean temperature for optimum germination and degree days) that elicited the most rapid and complete germination for each of the species used in these investigations. In the main, the mean optimal germination temperature requirement for the more geographically restricted species was slightly lower at 17.7 °C, but this was not significantly different to the mean T_opt_ for common species (18.8 °C) (df = 1, 111, *t* = 1.04, *p* = 0.302). Just under half of the collections had a day temperature requirement below 20 °C (mean 17.1 °C) with a mean night temperature of 12.7 °C. A similar number had day temperature preferences between 20–30 °C (mean 23.8 °C) and night temperature of 16.1 °C. Ten collections had day temperature preferences for germination above 30 °C (mean day temperature of 35 °C and mean night temperature of 23 °C). The lowest optimal mean temperature requirement (10 °C) was for seeds of *Banksia dryandroides* from the far south coast near Albany, however, this species does not currently have conservation status in Western Australia although it is restricted in its distribution. The requirement for alternating temperatures was low with most species showing a preference for small temperature fluctuations for optimum germination performance (common species mean 6.8 °C; restricted mean 6.3 °C) (Figure 2, Table 1. There were a number of outlier species that germinated optimally when there were large fluctuations in temperature, notably *Kunzea acicularis* (28 °C amplitude), but most species, for example in the genus *Banksia*, showed a preference for small fluctuations. The majority of *Eucalyptus* species preferred constant diurnal temperatures for optimal germination. Contour plots for each collection show the species-specific temperature requirements for observed germination success across the 49 cells of the temperature gradient plate (Figure S1).

**Figure 3:**
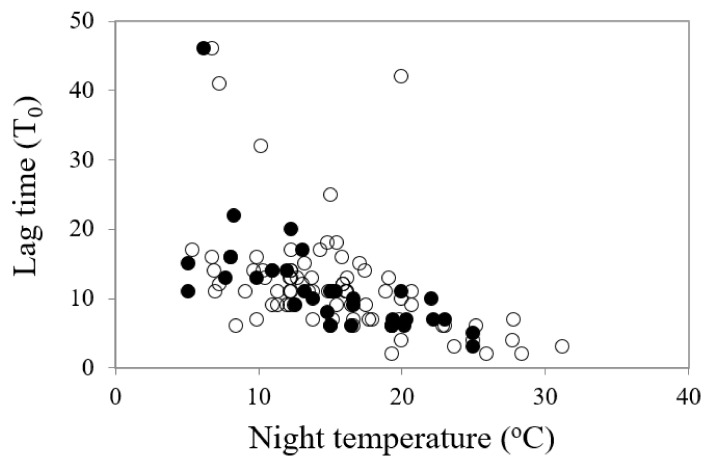
A comparison of the relationship between time to commence germination (lag time, T_0_) and night temperature for most rapid and highest germination in common (○) and restricted species (●) from southern Western Australia.

The General Linear Regression (GLM) analyses identified that all temperature parameters (mean, day, night and amplitude) had significant impacts on the onset of germination (lag time, T_0_) when all species were considered, but it was the night temperature that was the stronger driver of germination commencement (df = 1, 111, *F* = 38.61, *p* < 0.001) (Table 2). The lower the night temperature, the slower the onset of germination (Figure 3). Similarly, all temperature parameters had significant impacts on the reciprocal of time to 50% germination (germination rate, 1/T_50_), with one exception: the amplitude of diurnal temperature fluctuations was only significant for the restricted species and not for the common species (Table 2). All temperature parameters had significant impacts on mean time to germination. Kruskal–Wallis H tests recorded significant differences in percentage germination (df = 1, *H* = 1.657, χ^2^ probability = 0.034) and seed weight (df = 1, *H* = 4.342, *p* = 0.037) between the common and restricted species (higher germination and lower seed weight for restricted species) but no significant differences between the two categories of species in mean temperature for optimum germination, timing of germination (lag time, 1/_T50_ or MTG) or degree days for germination.

**Figure 4:**
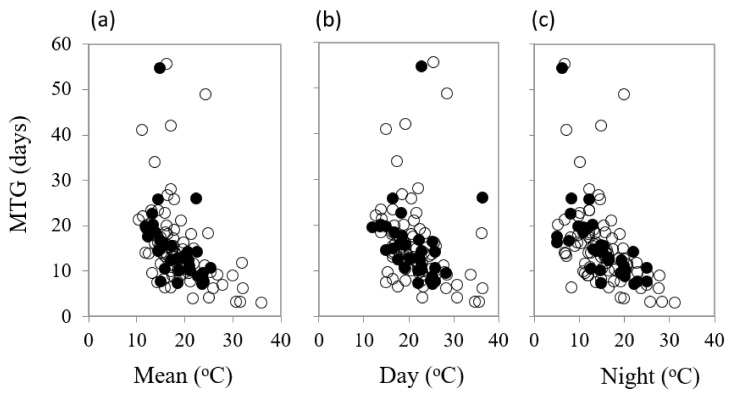
The impact of (**a**) mean diurnal temperature (**b**) day temperature and (**c**) night temperature for optimum germination on mean time to germination (MTG) in common (○) and restricted (●) species from southern Western Australia.

**Figure 5:**
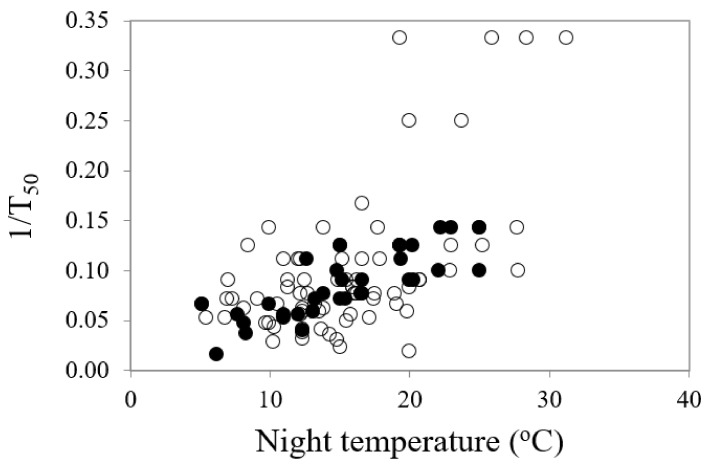
The relationship between mean optimum night temperature for germination and germination rate (inverse of time to 50% germination, 1/_T50_) in common (○) and restricted (●) species from southern Western Australia.

Mean temperature for optimum germination (T_opt_) had a significant influence on mean time to germination (MTG) (df = 1, 111, *F* = 20.68, *p* < 0.001) (Table 2). As various temperature parameters of the diurnal cycle increased, the mean time to final germination reduced, but there were some differences in the responses between common and restricted species (Figure 4a–c). Night temperature was also a highly significant (df = 1, 109, *F* = 59.75, *p* < 0.001) driver on time to 50% germination (germination rate, 1/T_50_), accounting for over one third of percentage variance in response. Restricted species showed a greater response to night-time temperature (Figure 5).

**Figure 6:**
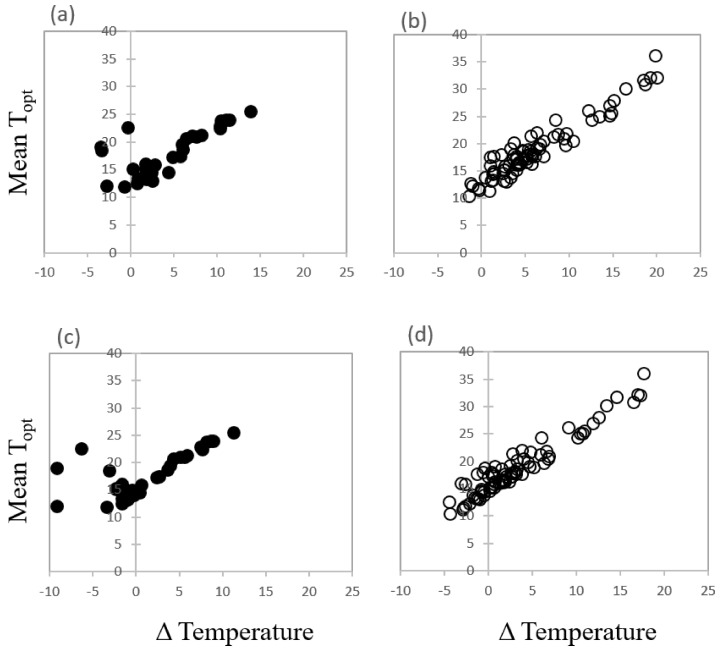
The difference (Δ) in observed mean temperature for optimum germination (empirical data) and current (1970–2000) temperature in the wettest quarter plotted against the mean temperature for optimum germination (T_opt_) for (**a**) restricted species and (**b**) common species. Fourteen collections are within 1 °C or below of the mean optimum temperatures for germination of the current wettest quarter. By 2061–2080, 50 collections are predicted to be within 1 °C or below threshold mean temperatures for germination in the wettest quarter (**c**) restricted species and (**d**) common species.

Overall, there was no relationship between the optimum temperature for most rapid and complete germination of each species and the mean annual temperature at seed collection sites. Neither was there any link between mean T_opt_ for germination and the mean temperature in the wettest quarter when seeds would be expected to germinate in this seasonally dry environment. However, there were species with mean temperature optima below or close to temperatures in the wettest quarter (Figure 6). For example, nine species *Banksia baxteri, B. dryandroides*, *B. meisneri* subsp. *ascendens*, *B. oreophila*, *Eucalyptus kruseana*, *E. nigrifunda*, *E.pimpiniana*, *E. jimberlanica*, and *Rhodanthe pyrethrum* showed a clear preference for optimum mean temperature for germination lower than the temperature experienced at their collection sites during the current wettest quarter of the year and this suite of species are shown left of the y axis line as negative temperature change (Figure 6a,b). A further four species, *Banksia aculeata*, *B. laevigata* subsp. *laevigata*, *B. sphaerocarpa*, and *Eucalyptus crispata* favoured mean temperature conditions very similar to, or within 1 °C of, temperatures already experienced in the wettest quarter of the year (these are centred around the y axis line). The remaining species preferred temperature conditions much higher than those recorded at their seed source sites and some displayed remarkably high thermal tolerance during the germination phase. When mean temperature conditions for optimum germination were plotted against forecast temperature values for the period 2061–2080 for the wettest quarter of the year, 50 collections showed a disparity between the empirical temperature data derived for optimum germination and the forecast temperature data (Figure 6c,d).

**Figure 7:**
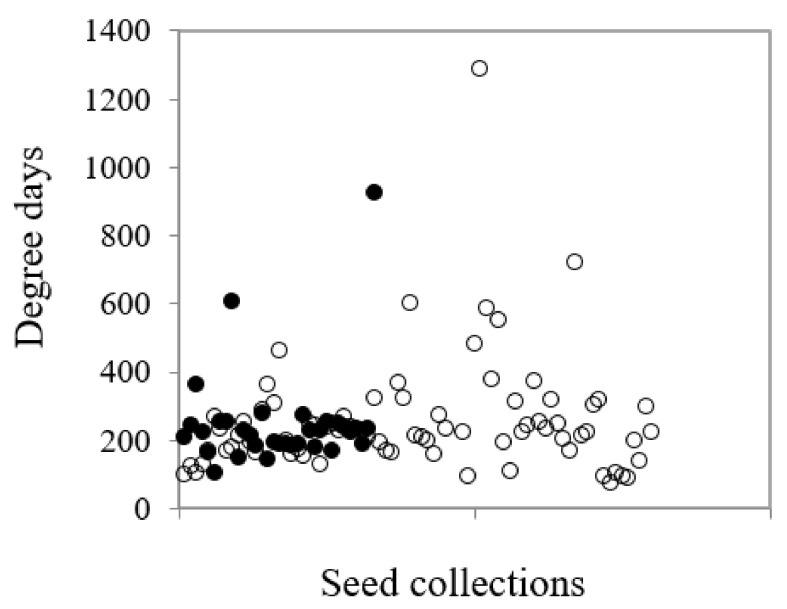
The relationship between common and restricted species for the degree days (as measured by mean temperature x time to 50% germination, T_50_) in common (○) and restricted (●) species from southern Western Australia.

Overall, degree days were a significant predictor of percentage germination across the species (df = 1,109, *F* = 45.04, *p* < 0.001). The suite of species with restricted distributions tended to have higher minimum and lower maximum degree days for optimal germination than common species (114–815 degree-days vs. 87–1186 degree-days) (Figure 7), but this was not a significant difference. Seed weight also impacted on germination (df = 1,111, *F* = 4.45, *p* = 0.037), however, it was the suite of restricted species that drove this result (df = 1,31, *F* = 17.41, *p* < 0.001). There was a significant negative relationship between seed weight (as measured by seeds per gram) and degree days for germination at T_opt_ but only for the restricted species (df = 1,31, *F* = 55.92, *p* < 0.001); the greater the seed weight, the larger the degree day value; however, this value signified both higher mean temperature with slower time to reach T_50_ as well as lower mean temperature and more rapid time to T_50_.

## 3. Discussion

### 3.1. Species Vulnerability

This paper provides an overview of the risk of rising temperatures facing the species-rich regional flora of southern Western Australia. Seeds of most species investigated were able to tolerate temperature extremes and successfully germinate above mean temperature conditions experienced in their current environment at a time when seeds would be expected to germinate (i.e., during the wettest quarter of the year). Although temperature is an important influence on seasonal timing of germination [49], for most species the temperature regime for optimal germination did not match a particular season. Finding parallels between germination temperatures and season of field emergence are not always easy. Modelling of these parameters is generally done in a controlled environment or in relation to fire-response [28,50]. Seal et al. [33] identified temperatures during the wettest quarter of the year as being the most significant climatic variable influencing seed response in a range of species in the family Cactaceae from the Americas. In this current series of investigations, the germination temperature optima of most species did not closely resemble the mean annual temperatures at collection sites, nor the current temperatures experienced during the winter wet. Around 88% of the species investigated for southern Western Australia had observed mean temperatures for optimum germination above the current temperature during the wettest quarter of the year. The presence of high physiological tolerance is an important attribute for species to track shifting climates [51] and these data lend some support to one of the major hypotheses of this investigation. Although these data may not be good predictors of current season of field emergence of these species, what has been demonstrated here is that the thermal tolerance for germination of a large portion of the 100+ native species from southern Western Australia is way beyond that of their realised thermal niches, albeit in the presence of unlimited moisture. The degree to which these species can tolerate temperature conditions outside their observed climatic locations may have a significant impact on their ability to persist in situ and avoid population extinction. However, the results indicate that a small number of species (12%) had optimum mean temperatures for germination slightly lower than the temperatures experienced at their seed source sites during the wettest quarter of the year, or else favoured mean temperature conditions very similar to, or within 1 °C of, temperatures already experienced in the wettest quarter of the year. This suggests that as local temperatures rise due to climatic change, these species may be reliant on germinating outside their optimum temperature conditions, and may experience a reduction in speed and/or success of germination, face a shift in timing of germination or at worst face regenerative failure. Indeed, when species mean temperature optima were plotted against forecast temperature in the wettest quarter for the period 2061–2080, more than 40% of species (50 collections) investigated were likely to be impacted, with germination in the future occurring under supra-optimal temperature conditions. From this perspective, it is possible to provide an overview of the vulnerability of a representative portion of the flora of southern Western Australia, albeit with no pattern. Generally speaking, it would appear that the majority of the plants of this seasonally dry region of southern Western Australia are not yet regenerating on the edge of their thermal tolerances, but many are likely to be towards the end of this century. However, in the absence of a geographic pattern in temperature optima for germination, there is little support for the hypothesis that species from northern sites would be less vulnerable to warming than those from southern sites. Four species of *Eucalyptus* inhabiting the transitional rainfall zone of southern Western Australia are forecast to experience a 3–7 °C increase in temperature in the wettest quarter by 2070 (BIOCLIM, [46]. These species are already germinating at temperatures below wet quarter means. Overnight temperatures in this more arid zone can be as low as 5 °C and occasionally fall below freezing. The most reliable rainfall occurs in winter, like that in the higher rainfall zone. These low overnight temperatures may indicate why these four *Eucalyptus* species have such low temperature optima. However, as the environment changes, the increase in wet quarter temperature will render these species especially vulnerable to germination failure as thermal thresholds are further surpassed. In the desert regions of Australia where heat wave extremes are becoming more frequent, leaf metabolism appears to be at threshold levels and plants are considered to already be living on the edge of their tolerances [52]. Indeed, more than 40% of the realised climatic niches of most *Eucalyptus* species from across Australia are considered to have ranges of less than 2 °C mean annual temperature, including 25% with less than 1 °C [53]. For the remaining species that were investigated from the southern Western Australia flora, most inhabit the high rainfall zone. Here, a number of *Banksia* species form part of a suite of species showing higher vulnerability to thermal conditions as they also experience a rise in wet quarter temperatures, and in particular *Banksia dryandroides* whose low germination temperature optimum makes this species highly vulnerable to regeneration failure. As forecasts for the region indicate increases in both day-time maximum and overnight minimum temperatures [54], it may be reasonable to assume that germination rates will decline, or else the timing of germination will shift seasonally. The latter scenario is more plausible and has been reported when modelling other floras e.g., see [27].

### 3.2. Temperature Preferences

One of the findings of this investigation was that the low phase of the diurnal cycle was more influential in driving germination timing, with species demonstrating slower rates of germination with lower night temperatures. However, few showed a preference for fluctuating temperatures; most showed a preference for constant temperatures, and quite a number had high germination regardless of the magnitude of the temperature fluctuations (see Figure S1). As previously mentioned, most studies using thermal time models only test constant temperatures or at best one diurnal temperature cycle for germination (e.g., [31,32,33] with few exceptions [34,35]. However, constant temperatures rarely occur in the natural environment [36]. Alternating temperatures allow seeds to sense seasonal shifts, depth of soil burial or gaps in vegetation [29], and in some species alternating temperatures can increase the proportion of seeds germinating, thereby substituting for a light requirement for germination [12]. Small seeded species are likely to respond better to fluctuating temperatures [55]. The requirements for diurnally fluctuating temperatures may be a characteristic of species from different habitats (e.g., wetland species [56]) or of different life forms (e.g., herbaceous species [57]) and it is often assumed that fluctuating temperatures are more favourable for germination [29]. However, much of the data arises from studies in the northern hemisphere and not necessarily from Mediterranean-type climates or from woody perennial species. This investigation of southern Western Australian species focused predominantly on woody perennials, most of which are assumed to have field emergence in late autumn or later in spring when temperatures show more constancy than during winter. Previous studies on this flora have demonstrated that constant temperatures provide adequate conditions for rapid germination [5].

Species responses were not grouped in any particular manner, though some small-seeded species displayed optimum germination under unusually high day-time temperatures (e.g., *Eucalyptus myriadena*, *Kunzea acicularis* and *Melaleuca preissiana*), but the majority did not. Small seeds that would germinate on, or very close to the surface of soil are likely to experience much higher temperatures than larger seeds germinating and emerging from greater depth. However, there was no relationship between temperatures for optimal germination and seed weights as some larger-seeded species like *Banksia seminuda* also had high optimal day-time temperatures for germination. The exceptionally high (>30 °C) mean temperatures for optimal germination performance for many species (e.g., *Eucalyptus erythrocorys*, *E. myriadena*, *Melaleuca preissiana*, *M. penicula* and *Neurachne alopecuroidea*) may indicate a propensity to germinate in autumn shortly after dispersal rather than in spring whilst soils are still warm but rainfall has commenced. Germination in response to higher temperatures may also be a gap-detection mechanism for seeds and regeneration may occur more readily on roadsides, cleared areas and after death of vegetation. Those species with lower (<13 °C) mean temperature optima (e.g., many of the *Banksia* species and *Eucalyptus jimberlanica* and *E. georgei* subsp. *fulgida*) may be reliant on germinating in spring when soils are cooler but still moist after winter rains.

### 3.3. Restricted Versus Common Species Response

There were only a few significant differences between the temperature responses of the common and restricted species. Principally the differences occurred in the association between day temperature and germination lag time, mean time to germination and the reciprocal of time to 50% germination. Germination responses in common species differed from those of the restricted species in several ways. The latter had a narrower temperature niche for germination than the former, with night-time temperatures more important than day or daily mean temperatures. In contrast, the suite of common species investigated demonstrated a greater response to mean temperature, with the amplitude of diurnal temperature fluctuations of no significance. Studies comparing germination responses of common and geographically restricted species, however, have generally report mixed results [58,59,60], and germination characteristics do not appear to explain rarity [61]. Reviewers have concluded that many trait patterns between rare and common species vary at spatial scales, examples showing that rare species had a higher minimum temperature for 50% germination, had no difference in germination rate or that rare species have less capacity for immediate germination [59].

### 3.4. Limitations

Studies into the seed biology of flowering plants focusing solely on a single driving force for germination are not without limitations. Despite temperature being one of the most important drivers of germination, germination cannot occur in the absence of moisture. Water stress influences both timing of germination and its success and was not accounted for during these investigations with seeds germinated under non-limiting moisture conditions. In addition, the artificial nature of laboratory experiments rarely simulates field scenarios and temperatures would unlikely remain unchanged over a period of 6 weeks as seasonal conditions alter perceptively over time. Importantly, a single collection of seeds made in one season from one site is not necessarily representative of a species across its geographic range [62] as seeds are known to exhibit phenotypic plasticity in their response to external stimuli [63] and these responses are likely to be affected by a changing climate [64]. Indeed, small differences in germination temperature optima did occur within the small number of species represented by multiple collections (for example see [10,65]. Most species investigated were long-lived woody perennials from several dominant families (e.g., Proteaceae and Myrtaceae). Although these life-forms are loosely reflected in the flora of southern Western Australia, an area dominated by forests, woodlands, shrubland and heath [66,67], there still remains a bias in the dataset and few annuals were investigated. Furthermore, a larger number of common species were investigated, potentially skewing the data. And finally, although storage conditions were similar across collections, seeds were stored for varying lengths of time and storage duration may have impacted on thermal requirements of collections by broadening the germination niche of some species. Storage time is known to affect germination success in some species with high levels of dormancy [68,69] and long periods of storage under conditions that promote after-ripening may result in seeds entering secondary dormancy or even losing viability [70].

Despite the above limitations, seed trait functional ecology research provides important data to improve climate model accuracy and help guide biodiversity programs aimed at conserving plant species in fire-prone environments in the face of a changing climate [71,72]. Germination is a critical stage in the life history of a plant and is an irreversible process: once a seed germinates it either establishes or dies. Warmer temperatures will not only impact on germination probabilities, but also affect seed development and seed traits such as seed mass, dormancy, seasonality of germination, seedling establishment, and soil and canopy seed bank survival [11,64]. Temperature cues for germination may also be influenced by the thermal memory of plants [73], increasing the complexity of identifying changing rate and thresholds for germination. In many human-impacted ecosystems, habitat fragmentation will also restrict colonisation as conditions for population persistence shift spatially and temporally. These complex webs of relationships between temperature and regenerative traits pose many challenges for predicting plant response to current and future climates [15]. Notwithstanding the many limitations outlined above, germination data can help reduce uncertainty in the management of the flora and in the choice of species for restoration and potential future outcomes. These findings suggest both winners and losers and these occur across both the common and restricted species in this regional flora. Many are quite ancient and have weathered climatic change in the past [67] and may well adapt to climatic change through environmentally-induced shifts in phenotype in the future [63]. Clearly, the smaller the range size and the narrower the germination temperature niche, the greater the likelihood of risk in the face of global change. Ensuring local persistence may be key to the continued survival of these species.

## 4. Methods

### 4.1. Species, Climate and Habitat

**Figure 8:**
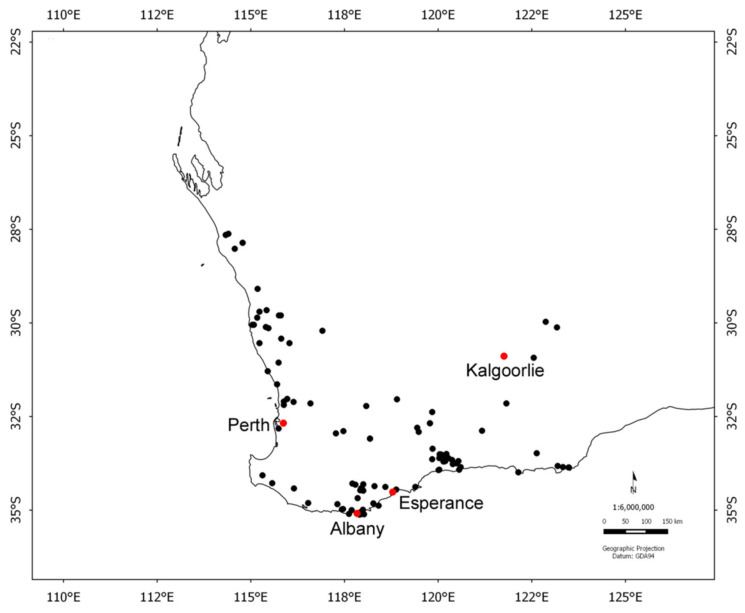
Locations of species investigated from southern Western Australia.

Seeds from the 102 species (113 collections) used to model the thermal requirements for germination were wild collected from sites across ~300 sq km in southern Western Australia, with a ~850 km north–south and ~1000 km east–west spread (Figure 8). Thirteen families and 23 genera were represented by these collections. Twenty-six collections originated from north of the Western Australian capital of Perth and the remaining 87 collections from south of the capital (31.95° S). Each collection represented a different site and species combination. Most of the species are classified as woody perennials and are endemic to the region. The majority are obligate seeders and are killed by fire and regenerate solely from seed. The number of seeds per gram ranged from 6 (*Callitris* and most *Banksia* species having larger seeds) to 44,000 for *Melaleuca preissiana*. Thirty-three species (33 collections) are restricted in their distributions in the region and are currently classified as rare or threatened [74] whilst 68 species (80 collections) have more widespread distributions and are deemed to be common within the landscape (see Table 3). These species occur principally in vegetation types such as forests, woodlands, shrublands, and heath. Species inhabiting this global hotspot experience a predominantly Mediterranean climate with most rain falling in the winter months and with summers characteristically dry. The average monthly minimum and maximum temperatures for each seed source site have been used as proxies for thermal tolerance and were obtained from WorldClim version 2.1, a set of global climate layers with a spatial resolution of approximately 1 km^2^ [75] (Table 4). The data for ‘current’ conditions were derived from 1970–2000 averages. Future projections (2061–2080) for the same climate variables were downloaded using climate model BCC-CSM2-MR using a high greenhouse gas emission scenario ssp585 (updated Representative Concentration Pathway, RCP 8.5) for the period based on high radiative forcing. This scenario reflects high energy demand and greenhouse gas emission without climate change policies [76]. This scenario is extreme but reflects a likely climate outcome given the current level of mitigation activity.

Seeds were collected from up to 50 individual maternal plants of each species at each seed source site over a period of 15 years between 2002 and 2017. After collection, seeds were extracted from fruits, dried, weighed and stored at 15 °C and 15% relative humidity or stored frozen at −20 °C when dried until their use in the experiments. The individual maternal collections of seeds of each species were bulked for germination purposes prior to use.

### 4.2. Experimental Design

A bi-directional temperature gradient plate (Model GRD1, Grant Instruments, Cambridge, UK) was used to provide seeds with a gradient of temperature combinations roughly between 5 and 40 °C (both constant and alternating) with a 12-h photoperiod. When the TGP gradient ran in a bi-directional mode, one-half of the cell grids received 12 h of light during the warm part of the diurnal cycle and the other half received 12 h of light during the cool part of the cycle. Four collections were germinated at each run of the TGP, providing 49 temperature combinations per collection with temperature magnitudes between 0 and 23 °C. Seeds were sown on 0.75% water agar in 30 mm plastic Petri dishes and monitored every 2–3 days for 6 weeks. Germinated seeds were removed and recorded. Seed numbers per dish ranged from 5 to 20 seeds depending on seed size and availability. At the end of the experiment, seeds with a hard, white endosperm were considered potentially viable; empty seeds were removed from the original count. Percentage germination was calculated as the percentage of seeds that germinated within the incubation period. No replication of temperature combinations was possible, and each seed was treated as an independent unit. This lack of replication is duly acknowledged; however, each species was represented by between ca. 490 and 980 seeds. Most species were non-dormant and required no pre-treatment for germination. Members of the Fabaceae with known physical dormancy [29] were pre-treated by removing a small portion of the hard water-impermeable seed coat with a scalpel prior to incubation. Other species with known dormancy mechanisms were treated accordingly (see Table 1). This same experimental design was used in previous studies [46,47,48].

The temperature parameters for germination (diurnal temperatures, their mean and amplitude) for each collection across the 49 incubation cells were logged and recorded. Germination percentage and rate measurements were calculated for each cell where germination occurred and included the lag time (i.e., days to first germination, T_0_), time to reach 50 percent of final germination (T_50_), and its reciprocal (1/T_50_, germination rate) and the mean time to germination (MTG days). MTG was calculated for the treatments that gave maximum germination using the equation: MTG = Σ (n × d)/N, where n is the number of seeds germinated between scoring intervals; d is the incubation period in days at that time point; and N is the total number of seeds germinated. From this dataset, only the information from the cell that elicited the most rapid and highest germination (termed optimum temperature for germination, T_opt_) for each collection was used in the analyses. In reporting the optimum thermal requirements for germination, I have ignored temporal patterns of germination as germination patterns were not compared within a species, rather between species. The mean of the diurnal temperatures was used as an indicator of season of germination as well as being a good index of biological activity [77]. Speed of germination is driven by accumulated temperature, or degree-days. In its most simplified form, degree-days are the sum of the average daily maximum and minimum temperatures over consecutive days (e.g., 12 h at 10 °C and 12 h at 25 °C equate to a mean of 17.5 °C multiplied by the germination rate in days). In this research, I calculated degree days as the mean diurnal temperature multiplied by the time to 50% germination in days. The concept of degree days is based on the hypothesis that the rate of germination increases in a linear manner with temperature to a point considered to be the optimal temperature for germination [78]. Further increases in temperature can reduce both the germination rate and the germination capacity to zero and would be considered as supra-optimal for germination. Seed weights were determined based on three replicates of at least 50 seed per species, and the number of seeds per gram was subsequently calculated for each collection.

### 4.3. Data Analysis

General Linear Regression (GLM) was used to evaluate how the optimum temperature regime (diurnal temperatures, mean and magnitude) for the most rapid and highest germination for each collection affected the rate of seed germination (T_0_, 1/T_50_ and MTG). These three features of germination rate were used as dependent variables, whilst the temperature values (diurnal temperatures, average of day/night temperatures, and the magnitude of the diurnal temperature range) for the cell on the gradient plate providing the most rapid and complete germination were used as the independent variables in the regression analyses. The effects of diurnal temperature variability on germination rate can be non-linear. To overcome these possible non-linear responses, the squares of temperature variables were included in each model. Kruskal–Wallis H tests were used to determine differences in germination data (timing, percentage and temperature conditions) between the common and restricted species. Modified degree days were calculated as the mean temperature for optimum germination x days to 50% germination. GLM’s were used to assess the impact of degree days and seed weights (as seeds per gram) on germination capacity. Seed weight can have a strong effect on germination [29]. Current and future climate data were extracted from WorldClim, a set of global climate layers with a spatial resolution of approximately 1 km^2^ [75]. Contour plots illustrating the complete germination data for each collection were created in Origin 9.1.0 (Origin Lab Corporation, Massachusetts, USA). Statistical analyses were performed in GENSTAT 18th edn (VSN International, Hemel Hempstead, UK).

### 4.4. Ethical Statement

Seeds from all restricted species (rare and threatened) were collected by the author or her colleagues from the Department of Biodiversity, Conservation and Attractions with relevant flora licences issued under the Western Australian *Biodiversity Conservation Act 2016* or prior to 2016 under the *Wildlife Conservation Act 1950*. Herbarium voucher specimens for each collection have been lodged at the Perth Herbarium and details can be found at https://florabase.dpaw.wa.gov.au/.

## 5. Conclusions

Germination is a complex adaptive trait that determines the establishment of the next generation of plants and contributes to population persistence. Research into seed response to climatic factors provides important information for biodiversity conservation and for the in situ and ex situ management of flora. Despite the considerable advancement in seed knowledge, seed traits and functions remain little explored topics and there are still a surprising number of knowledge gaps. The relationship between seed traits and functions is more advanced in some areas (e.g., crop and weed research), but we need to extend our approach to more vegetation systems including native species beyond the agricultural sphere. Identifying species with high thermal tolerance is one thing but we should investigate the genetic, molecular, biochemical, and physiological mechanisms that underlie these temperature preferences. We may wish to ask whether it is true that temperature requirements do confer an ecological advantage to seedlings generated and comprehensively investigate this question across broad geo-climatic and ecological gradients. Would conducting seed ageing experiments provide clues to the thermal tolerances of seed? How do temperature requirements for germination and fire interact, particularly in Mediterranean-climate ecosystems? Any research undertaken needs to be taken into the real world through field experimentation and subsequent use in restoration. In addition, do we really know how to use this data in decision making? If climate does function as an evolutionary force influencing the thermal opportunities for germination, we might be able to see natural selection in action during environmental change and act accordingly. We can expect this to be a challenge.

## Figures and Tables

**Table 1:** Summary data for germination parameters for 113 collections (102 species). C = common species (*n* = 80); R = restricted species (*n* = 33).

		Mean	Min	Max	Median	SD
**Germination (%)**	**C**	95.4	8.0	100.0	100.0	14.41
	**R**	98.4	58.0	100.0	100.0	7.46
**T_0_ (lag time) (days)**	**C**	11.9	2.0	46.0	11.0	7.93
	**R**	11.7	3.0	46.0	11.0	7.25
**T_50_ (days)**	**C**	14.3	3.0	53.0	13.0	8.69
	**R**	14.8	7.0	62.0	13.0	9.84
**diff T_0_ to T_50_ (days)**	**C**	3.2	0	21	2	4.17
	**R**	3.1	0	16	3	3.03
**1/T_50_**	**C**	0.098	0.019	0.333	0.0769	0.069
	**R**	0.083	0.016	0.143	0.0769	0.032
**MTG (days)**	**C**	15.5	3.0	55.6	13.8	9.49
	**R**	15.6	7.0	54.7	14.2	8.67
**Day temperature (°C)**	**C**	22.2	12.8	41.5	20.9	6.19
	**R**	21.1	12.0	36.4	22.1	5.06
**Night temperature (°C)**	**C**	15.4	5.4	31.2	15.0	5.58
	**R**	14.9	5.1	25.0	14.8	5.59
**Amplitude (°C)**	**C**	6.8	0.4	22.6	5.9	4.87
	**R**	6.3	0.0	28.1	4.0	6.32
**Mean Topt (°C)**	**C**	18.8	10.4	36.1	17.6	5.34
	**R**	17.7	11.8	25.5	17.2	4.06
**Degree days for T_50%_**	**C**	255	75	1288	225	169
	**R**	247	105	924	226	147

**Table 2:** Regression coefficients and adjusted R^2^ values for regression models describing the relationship between germination rate (lag time, 1/T_50_ and MTG) and temperature parameters (diurnal temperatures, amplitude of temperature fluctuations [Δ] and mean daily temperature) for 113 collections (102 species). MTG = mean time to germination; 1/T_50_ = reciprocal of time to achieve 50% germination; C = common species (*n* = 80); R = restricted species (*n* = 33).

	Species	Day Temperature		Night Temperature		Δ Temperature		Mean Temperature	
		Regression Coefficient	R^2^	Regression Coefficient	R^2^	Regression Coefficient	R^2^	Regression Coefficient	R^2^
lag	all	−0.5950 *	0.047	−1.308 ***	0.254	0.115 *	0.053	−1.298 ***	0.153
	common	−0.3670 *	0.065	−1.067 ***	0.198	0.0110	0.000	−1.286 ***	0.156
	restricted	−2.4100	0.002	−1.797 ***	0.378	0.5060 *	0.182	−1.310	0.093
1/T50	all	0.0003 ***	0.251	0.0078 **	0.423	0.0038	0.009	0.0080 ***	0.404
	common	0.0015 ***	0.281	0.0108 ***	0.417	0.0061	0.000	0.0082 ***	0.399
	restricted	0.0173 *	0.252	0.0002 ***	0.602	−0.0012 *	0.123	0.0055 ***	0.331
MTG	all	−0.683 *	0.045	−1.2060 ***	0.229	0.1250 *	0.045	−1.282 ***	0.144
	common	−0.300 *	0.055	−0.7450 ***	0.169	0.0330	0.000	−1.000 **	0.130
	restricted	−2.910	0.034	−2.1800 ***	0.389	0.0547 **	0.154	−2.340 **	0.137

* *p* < 0.05, ** *p* < 0.01, *** *p* < 0.001.

**Table 3:** List of species investigated, including family, endemic and obligate seeder status, conservation ranking, their provenance and IBRA location. Seeds of most species were non-dormant seeds; however, those that displayed dormancy were treated according to known protocols (see below *#^).

Species	Family	Cons.code	Endemic	Obligate Seeder	Provenance	IBRA
Acacia besleyi *	Fabaceae	P1	Y	Y	Ravensthorpe	ESP
Acacia bifaria *	Fabaceae	P3	Y	Y	Ravensthorpe	ESP
Acacia disticha *	Fabaceae	-	Y	Y	Ravensthorpe	ESP
Acacia durabilis *	Fabaceae	-	Y	Y	Ravensthorpe	ESP
Acacia heterochroa *	Fabaceae	-	Y	Y	Ravensthorpe	ESP
Acacia ophiolithica *	Fabaceae	-	Y	Y	Ravensthorpe	ESP
Acacia pinguiculosa *	Fabaceae	-	Y	Y	Ravensthorpe	ESP
*Allocasuarina hystricosa*	Allocasuarinaceae	P4	Y	N	Ravensthorpe	ESP
*Anigozanthus manglesii* subsp. *Quadrans #*	Haemodoraceae	-	Y	Y	Enneaba	GES
*Banksia aculeata*	Proteaceae	P2	Y	Y	Stirling Range NP	ESP
*Banksia ashbyi*	Proteaceae	-	Y	Y	Ajana	GES
*Banksia attenuata*	Proteaceae		Y	N	Brighton	SWA
*Banksia attenuata*	Proteaceae	-	Y	N	Carnamah-Eneabba	GES
*Banksia attenuata*	Proteaceae	-	Y	N	Kalbarri NP	GES
*Banksia baueri*	Proteaceae	-	Y	Y	Stirling Range NP	ESP
*Banksia baueri*	Proteaceae	-	Y	Y	Fitzgerald River NP	ESP
*Banksia baueri*	Proteaceae	-	Y	Y	Tarin Rock NR	AVW
*Banksia baxteri*	Proteaceae	-	Y	Y	Stirling Range NP	ESP
*Banksia benthamiana*	Proteaceae	P4	Y	Y	Nudagong East Rd	AVW
*Banksia blechnifolia*	Proteaceae	-	Y	Y	Fitzgerald	ESP
*Banksia brownii*	Proteaceae	DRF	Y	Y	Vancouver Peninsula	ESP
*Banksia burdettii*	Proteaceae	-	Y	Y	Agaton Rd Reserve	GES
*Banksia caleyi*	Proteaceae	-	Y	Y	Stirling Range NP	ESP
*Banksia coccinea*	Proteaceae	-	Y	Y	Gull Rock NP	ESP
*Banksia dryandroides*	Proteaceae	-	Y	Y	Cheyne Rd NR	ESP
*Banksia grandis*	Proteaceae	-	Y	N	Marbellup	ESP
*Banksia hookeriana*	Proteaceae	-	Y	Y	Kalbarri NP	GES
*Banksia laevigata* subsp. *laevigata*	Proteaceae	P4	Y	Y	Ravensthorpe Range	ESP
*Banksia lanata*	Proteaceae	-	Y	Y	Badgingarra	GES
*Banksia laricina*	Proteaceae	-	Y	Y	Moore River NP	GES
*Banksia lemanniana*	Proteaceae	-	Y	Y	Ravensthorpe Range	ESP
*Banksia leptophylla* var. *mellitica*	Proteaceae	-	Y	Y	Coorow-Greenhead	GES
*Banksia lindleyana*	Proteaceae	-	Y	Y	Binnu West	GES
*Banksia media*	Proteaceae	-	Y	Y	Cape Arid NP	ESP
*Banksia meisneri* subsp. *ascendens*	Proteaceae	P4	Y	Y	Scott River	SWA
*Banksia nutans*	Proteaceae	-	Y	Y	Cape Arid NP	ESP
*Banksia occidentalis*	Proteaceae	-	Y	Y	Hay River	ESP
*Banksia oreophila*	Proteaceae	-	Y	Y	Hamilla Hills	ESP
*Banksia petiolaris*	Proteaceae	-	Y	Y	Jerdacuttup	ESP
*Banksia pilostylis*	Proteaceae	-	Y	Y	Springdale Rd	ESP
*Banksia pilostylis*	Proteaceae	-	Y	Y	Mt Howick	ESP
*Banksia praemorsa*	Proteaceae	-	Y	Y	Torndirrup NP	ESP
*Banksia prionotes*	Proteaceae	-	Y	Y	Carnamah-Eneabba	GES
*Banksia prionotes*	Proteaceae	-	Y	Y	Kalbarri NP	GES
*Banksia pulchella*	Proteaceae	-	Y	Y	Cape Arid NP	ESP
*Banksia quercifolia*	Proteaceae	-	Y	Y	Rudgyard NR	ESP
*Banksia scabrella*	Proteaceae	P4	Y	Y	Burma Rd NR	GES
*Banksia sceptrum*	Proteaceae	-	Y	Y	Ajana	GES
*Banksia seminuda*	Proteaceae	-	Y	Y	Manjimup	SWA
*Banksia solandri*	Proteaceae	P2	Y	Y	Stirling Range NP	ESP
*Banksia speciosa*	Proteaceae	-	Y	Y	Cape Arid NP	ESP
*Banksia sphaerocarpa*	Proteaceae	-	Y	N	Tarin Rock NR	AVW
*Banksia telmateiae*	Proteaceae	-	Y	Y	Jurien	GES
*Banksia verticillata*	Proteaceae	DRF	Y	Y	Torndirrup NP	ESP
*Banksia victoriae*	Proteaceae	-	Y	Y	Ajana	GES
*Banksia violaceae*	Proteaceae	-	Y	Y	Munglinup	ESP
*Banksia violaceae*	Proteaceae	-	Y	Y	Tarin Rock NR	ESP
*Beaufortia orbifolia*	Myrtaceae		Y	Y	Ravensthorpe	ESP
*Borya sphaerocephela*	Boryaceae		Y	N	Perth Hills	SWA
*Callitris pyramidalis*	Cupressaceae		Y	N	Camel Lake SRNP	ESP
*Calothamnus gracilis*	Myrtaceae		Y	N	Chillinup	ESP
*Calothamnus roseus*	Myrtaceae	P2	Y	Y	Ravensthorpe	ESP
*Carex tereticaulis*	Cyperaceae	P1	Y	Y	Harvey River	SWA
Daviesia megacalyx *	Fabaceae	DRF	Y	Y	Ravensthorpe	ESP
*Eucalyptus aequiperta*	Myrtaceae		Y	N	Narrogin	AVW
*Eucalyptus argutifolia*	Myrtaceae	DRF	Y	N	Seabird	SWA
*Eucalyptus burdettiana*	Myrtaceae	DRF	Y	Y	FRNP	ESP
*Eucalyptus calcicola* subsp. *unita*	Myrtaceae	P4	Y	N	West Cape Howe	ESP
*Eucalyptus cernua*	Myrtaceae		Y	Y	Ravensthorpe Range	ESP
*Eucalyptus conferruminata*	Myrtaceae		Y	Y	Albany	ESP
*Eucalyptus crispata*	Myrtaceae	DRF	Y	N	Bunney Rd	AVW
*Eucalyptus desmondensis*	Myrtaceae	P4	Y	Y	Ravensthorpe Range	ESP
*Eucalyptus erythrocorys*	Myrtaceae		Y	N	Coorow-Greenhead	GES
*Eucalyptus georgei* subsp. *fulgida*	Myrtaceae	P4	Y	Y?	Forrestiana	MAL
*Eucalyptus insularis*	Myrtaceae	DRF	Y	Y	Cape le Grande	ESP
*Eucalyptus jimberlanica*	Myrtaceae	P1	Y	N	Jimberlana Hill	COO
*Eucalyptus kondininensis*	Myrtaceae		Y	Y?	Narrogin	AVW
*Eucalyptus kruseana*	Myrtaceae	P4	Y	N	Cardunia Rocks	COO
*Eucalyptus livida*	Myrtaceae		Y	N	Ironcaps	MAL
*Eucalyptus marginata*	Myrtaceae		Y	N	Wandi	SWA
*Eucalyptus megacarpa*	Myrtaceae		Y	N	SRNP	ESP
*Eucalyptus megacarpa*	Myrtaceae		Y	N	Torndirrup	ESP
*Eucalyptus megacornuta*	Myrtaceae		Y	Y	Ravensthorpe	ESP
*Eucalyptus myriadena*	Myrtaceae		Y	N	Lake Varley	MAL
*Eucalyptus newbeyi*	Myrtaceae	P3	Y	Y	Bremer Bay	ESP
*Eucalyptus nigrifunda*	Myrtaceae	P4	Y	Y?	Mt Dennis	COO
*Eucalyptus nutans*	Myrtaceae	DRF	Y	Y	Bremer Bay	ESP
*Eucalyptus pimpiniana*	Myrtaceae	P3	Y	N	Ponton Creek	COO
*Eucalyptus preissiana* subsp. *lobata*	Myrtaceae	P4	Y	N	Starvation Boat Harbour	ESP
*Eucalyptus rugulata*	Myrtaceae	P4	Y	Y	Ironcaps	MAL
*Eucalyptus spathulata* subsp. *salina*	Myrtaceae	P3	Y	Y	Corrigin	AVW
*Eucalyptus staeri*	Myrtaceae		Y	N	Cheyne Beach	ESP
*Eucalyptus stoatei*	Myrtaceae	P4	Y	Y	Ravensthorpe Range	ESP
*Eucalyptus subtilis*	Myrtaceae		Y	N	Lake King	AVW
*Eucalyptus talyuberlup*	Myrtaceae		Y	Y	SRNP	ESP
Isopogon Ravensthorpe *	Proteaceae		Y	N	Ravensthorpe	ESP
*Kunzea acicularis ^*	Myrtaceae	DRF	Y	Y	Ravensthorpe	ESP
*Leptospermum incanum ^*	Myrtaceae		Y	Y	Peak Charles	ESP
*Melaleuca penicula*	Myrtaceae		Y	?	Ravensthorpe	ESP
*Melaleuca preissiana*	Myrtaceae		Y	Y	Jandakot	SWA
*Melaleuca stramentosa*	Myrtaceae		Y	Y	Ravensthorpe Range	ESP
*Neurachne alopecuroidea*	Poaceae		N	N	Tootbardie	AVW
*Neurachne alopecuroidea*	Poaceae		N	N	Perth Hills	SWA
*Neurachne alopecuroidea*	Poaceae		N	N	Narrogin	AVW
*Neurachne alopecuroidea*	Poaceae		N	N	Cocanarup	ESP
*Podolepis aristata*	Asteraceae		Y	Y	Cairn Hill	GES
*Regelia ciliata*	Myrtaceae		Y	Y	Dobaderry Swamp	SWA
*Rhodanthe pyrethrum*	Asteraceae		Y	Y	Brixton St	SWA
*Stylidium scandens*	Stylidiaceae	-	Y	Y	Mt Lindesay	WAR
*Xanthorrhoea preissii*	Xanthorrhoeaceae		Y	N	Brookton	SWA
*Xerochrysum macranthum*	Asteraceae		Y	Y	Porongurups	ESP
*Xyris lacera*	Xyridaceae		Y	Y	Beardwood Rd	WAR
*Xyris maxima*	Xyridaceae	P2	Y	Y	Spearwood Swamp	WAR

DRF = Declared Rare; P1-P4 = Data deficient species. IBRA = Interim Biogeographic Regions of Australia (www.environment.gov.au/land/nrs/science/ibra). AVW = Avon Wheat belt; COO = Coolgardie; ESP = Esperance Sandplains; GES = Geraldton Sandplains; MAL = Mallee; SWA = Swan Coastal Plain; WAR = Warren. * seed manually scarified prior to incubation. # seed heat shock treated for 3 hr at 100 °C. ^ seed soaked in 10% diluted solution of smoked water as Regen 2000 Smokemaster^®^ for 24 hr then rinsed in DI water.

**Table 4:** Summary information for 113 collections (102 species). MAP = mean annual precipitation; MAT = mean annual temperature C = common species (*n* = 80); R = restricted species (*n* = 33).

	Latitude		Longitude		Current MAP (mm)		Current MAT (°C)		MAP (2061–2080)		MAT (2061–2080)		Current Temperature in Wet Quarter (°C)		Temperature in Wet Quarter (2061–2080) (°C)		Seeds Per Gram	
	C	R	C	R	C	R	C	R	C	R	C	R	C	R	C	R	C	R
**Mean**	32.7	33.0	118.0	119.1	535.1	517.9	16.9	16.7	442.9	431.7	19.6	19.3	12.7	13.6	15.5	16.4	1912	1472
**Min**	27.6	29.1	114.3	115.2	301.0	238.0	13.8	14.3	272.0	233.0	16.5	16.6	9.9	10.2	13.3	14.0	6	9
**Max**	35.1	35.1	123.5	123.2	1126.0	1035.0	20.0	19.6	913.0	831.0	23.9	22.5	16.4	22.9	19.1	28.8	44,000	11,170
**Median**	33.6	33.6	118.0	119.8	460.0	415.0	16.3	16.2	384.0	346.0	19.0	18.7	12.4	12.7	15.0	15.3	76	633
**SD**	2.07	1.68	2.34	2.29	212.2	239.3	1.87	1.44	162.6	181.4	2.032	1.689	1.57	3.02	1.50	3.56	6007	2652

## Supplementary Materials

The following are available online at https://www.mdpi.com/2223-7747/9/6/796/s1, Figure S1: Contour plots for observed data for seeds of 113 collections. Points of equal percentage germination are connected by germination isopleths. The gradation in colours from dark (black—100%) to light (white—0%) represent decreasing percentage germination. The contour lines within each plot represent mean time to germination at various levels of germination. Constant temperatures are presented on the diagonal line from the bottom-left corner of the diagrams (lowest temperature c. 5 °C) to the top-right corner (maximum temperature c. 40 °C). All points above and below the diagonal line represent alternating temperature regimes, with greatest amplitude at the top-left and bottom-right corners of each graph. The diagonal line from bottom left to top right corner of each plot signifies the divide between diurnal cycles that have light during the warmer day regime (bottom right section) and dark during the warmer day regime (top left section). Note: The plot for *Stylidium scandens* was not displayed due to poor germination (<10%).
