# Supplementary material for: Thermal Requirements Underpinning Germination Allude to Risk of Species Decline from Climate Warming
Source: Plants (Basel). 2020 Jun 25;9(6):796. doi: 10.3390/plants9060796 (PMC7355932; doi:10.3390/plants9060796)
Supplement: Supplementary file 1 [file plants-09-00796-s001.pdf]

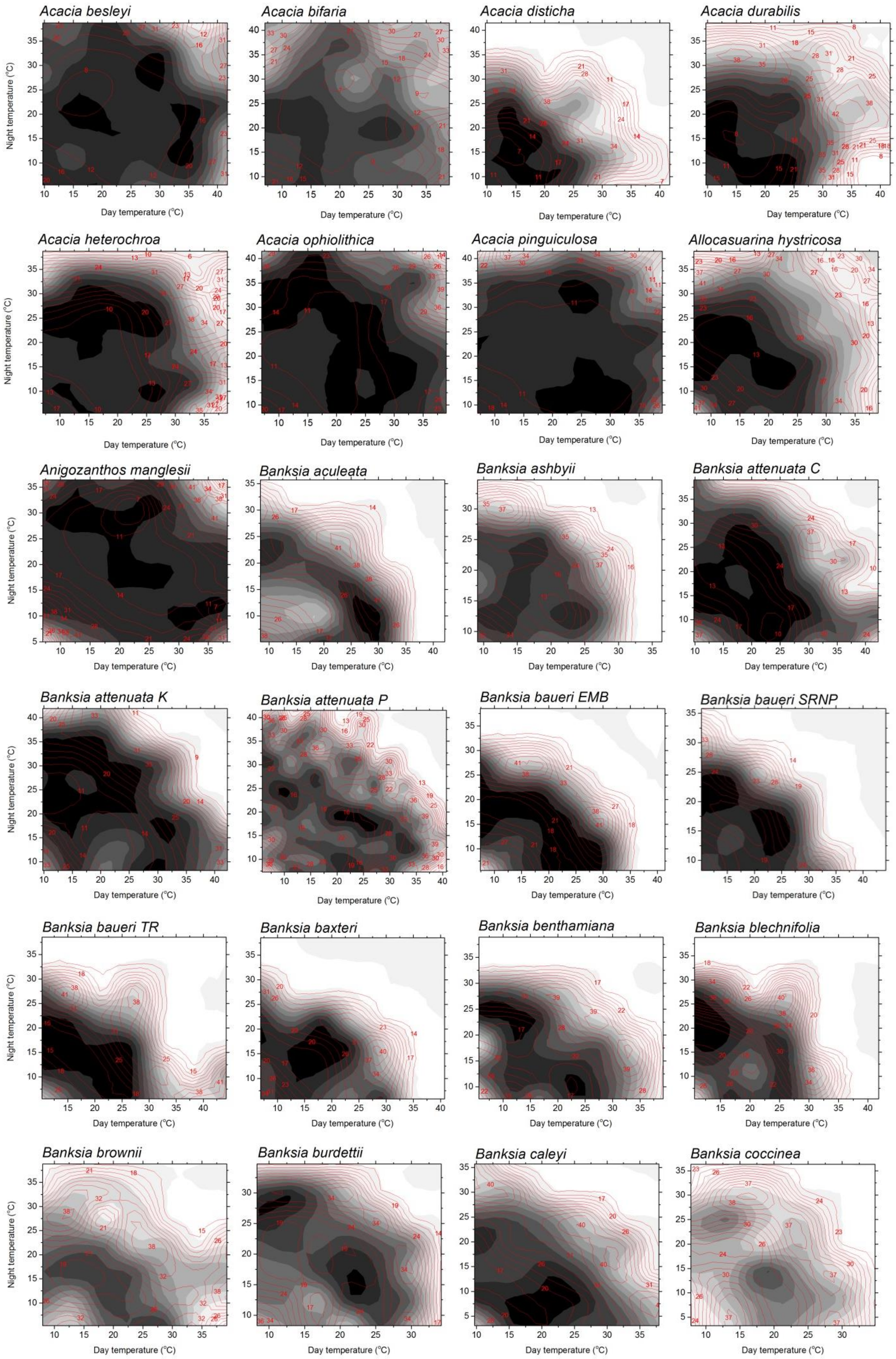
Acacia besleyi
Acacia bifaria
Acacia disticha
Acacia durabilis
Acacia heterochroa
Acacia ophiolithica
Acacia pinguiculosa
Allocasuarina hystricosa
Anigozanthos manglesii
Banksia aculeata
Banksia ashbyii
Banksia attenuata C
Banksia attenuata K
Banksia attenuata P
Banksia baueri EMB
Banksia baueri SRNP
Banksia baueri TR
Banksia baxteri
Banksia benthamiana
Banksia blechnifolia
Banksia brownii
Banksia burdettii
Banksia caleyi
Banksia coccinea
Night temperature (°C)
Day temperature (°C)

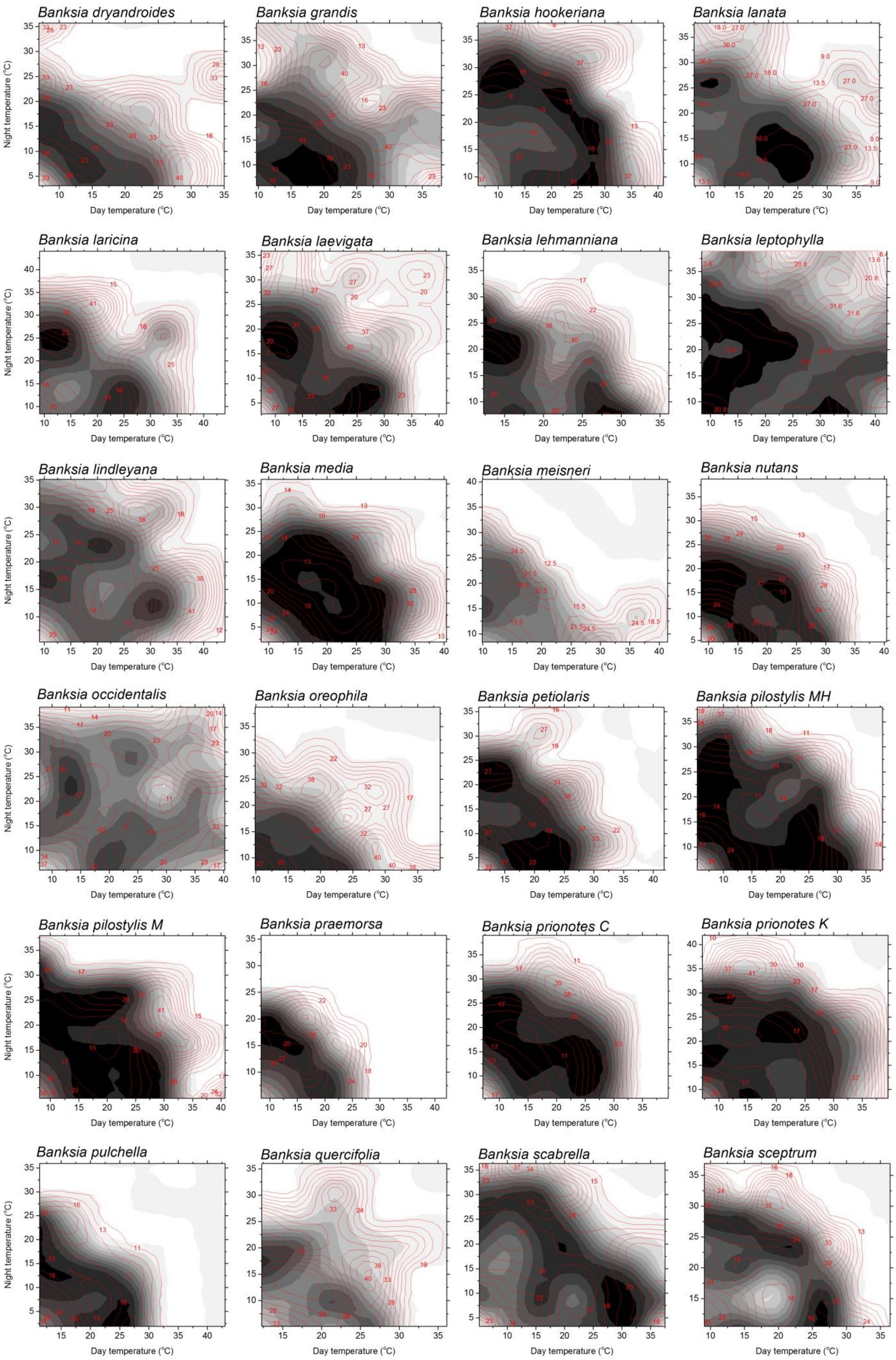
Banksia dryandroides
Banksia grandis
Banksia hookeriana
Banksia lanata
Banksia laricina
Banksia laevigata
Banksia lehmanniana
Banksia leptophylla
Banksia lindleyana
Banksia media
Banksia meisneri
Banksia nutans
Banksia occidentalis
Banksia oreophila
Banksia petiolaris
Banksia pilostylis MH
Banksia pilostylis M
Banksia praemorsa
Banksia prionotes C
Banksia prionotes K
Banksia pulchella
Banksia quercifolia
Banksia scabrella
Banksia sceptrum
Night temperature (°C)
Day temperature (°C)

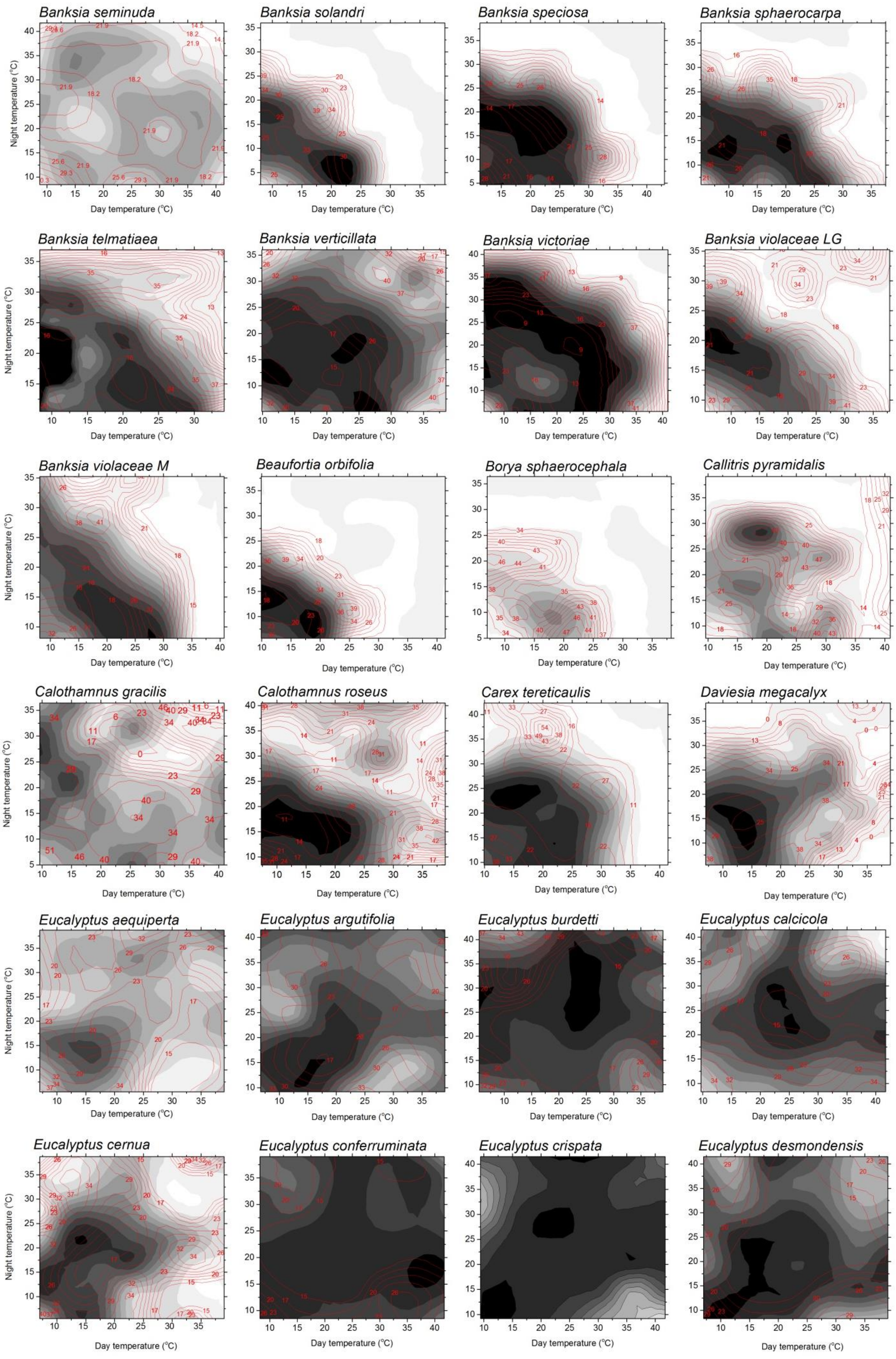

Banksia seminuda
Banksia solandri
Banksia speciosa
Banksia sphaerocarpa
Banksia telmatiaea
Banksia verticillata
Banksia victoriae
Banksia violaceae LG
Banksia violaceae M
Beaufortia orbifolia
Borya sphaerocephala
Callitris pyramidalis
Calothamnus gracilis
Calothamnus roseus
Carex tereticaulis
Daviesia megacalyx
Eucalyptus aequiperta
Eucalyptus argutifolia
Eucalyptus burdetti
Eucalyptus calcicola
Eucalyptus cernua
Eucalyptus conferruminata
Eucalyptus crispata
Eucalyptus desmondensis
Night temperature (°C)
Day temperature (°C)

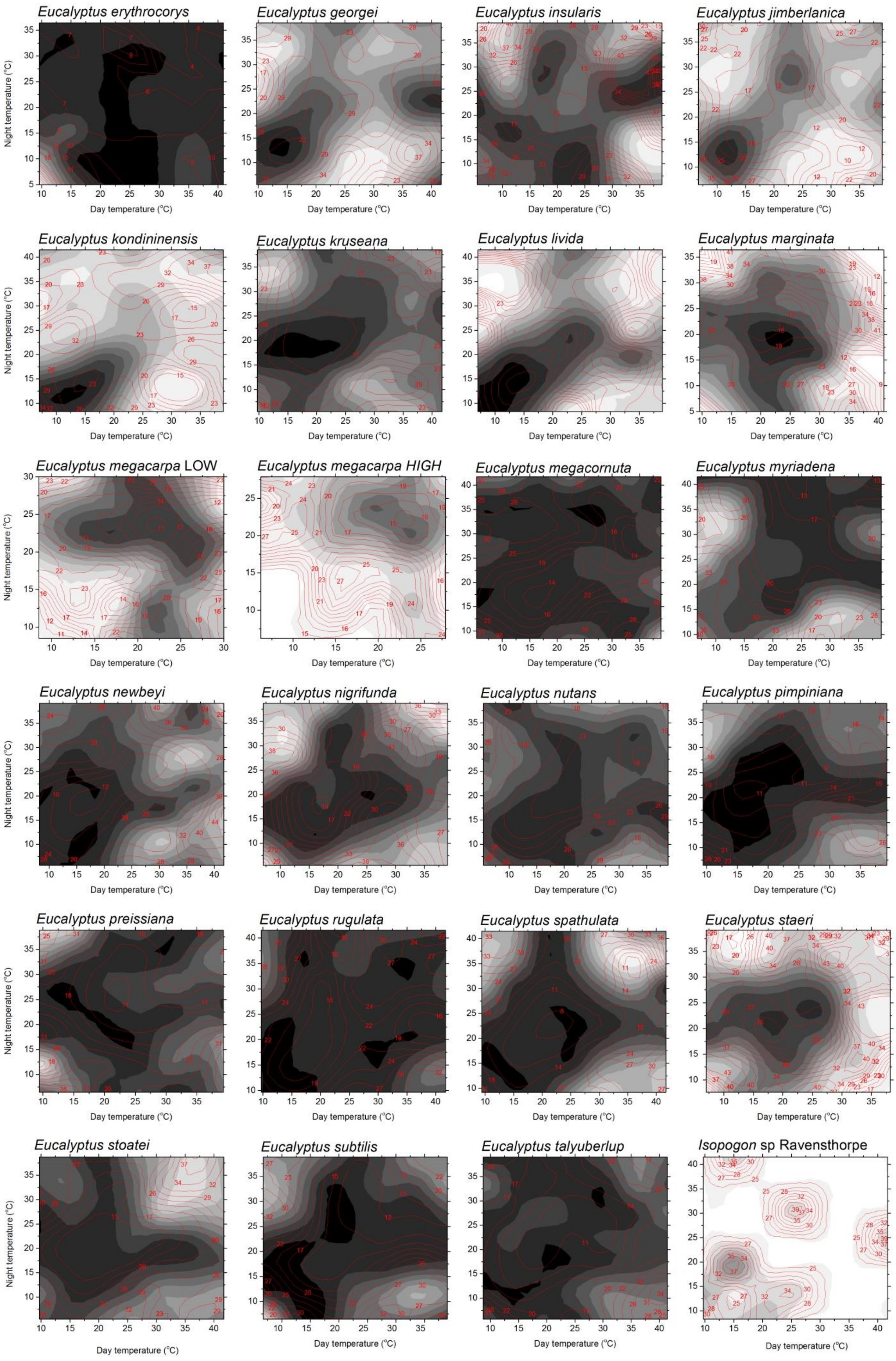
Eucalyptus erythrocorys
Eucalyptus georgei
Eucalyptus insularis
Eucalyptus jimberlanica
Eucalyptus kondininensis
Eucalyptus kruseana
Eucalyptus livida
Eucalyptus marginata
Eucalyptus megacarpa LOW
Eucalyptus megacarpa HIGH
Eucalyptus megacornuta
Eucalyptus myriadena
Eucalyptus newbeyi
Eucalyptus nigrifunda
Eucalyptus nutans
Eucalyptus pimpiniana
Eucalyptus preissiana
Eucalyptus rugulata
Eucalyptus spathulata
Eucalyptus staeri
Eucalyptus stoatei
Eucalyptus subtilis
Eucalyptus talyuberlup
Isopogon sp Ravensthorpe
Night temperature (°C)
Day temperature (°C)

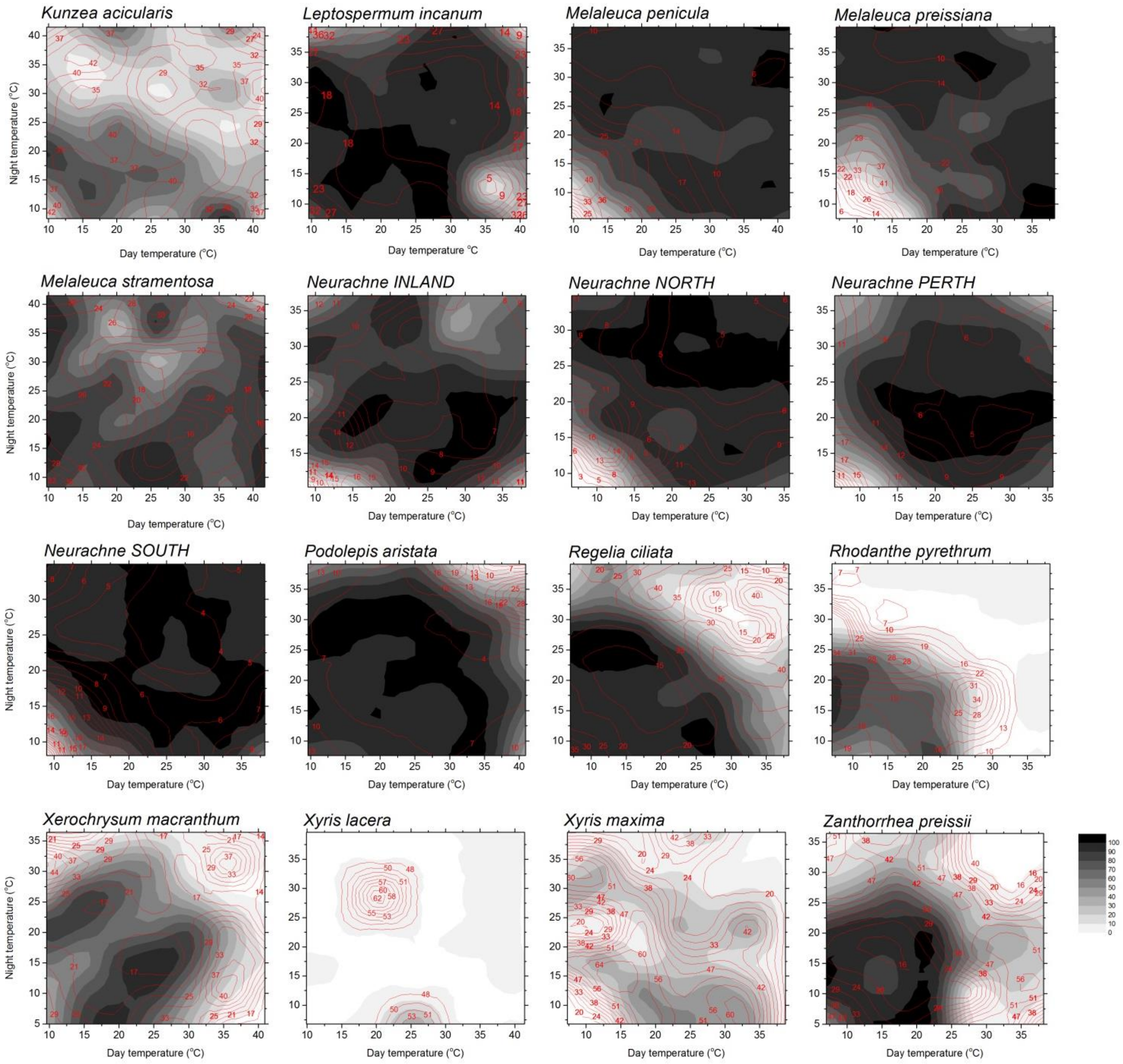

Figure S1. Contour plots for observed data for seeds of 113 collections. Points of equal percentage germination are connected by germination isopleths. The gradation in colours from dark (black - 100%) to light (white - 0%) represent decreasing percentage germination. The contour lines within each plot represent mean time to germination at various levels of germination. Constant temperatures are presented on the diagonal line from the bottom-left corner of the diagrams (lowest temperature c. 5ºC) to the top-right corner (maximum temperature c. 40ºC). All points above and below the diagonal line represent alternating temperature regimes, with greatest amplitude at the top-left and bottom-right corners of each graph. The diagonal line from bottom left to top right corner of each plot signifies the divide between diurnal cycles that have light during the warmer day regime (bottom right section) and dark during the warmer day regime (top left section). Note: The plot for *Stylidium scandens* was not displayed due to poor germination (<10%).
